# Pirfenidone increases transverse tubule length in the infarcted rat myocardium

**DOI:** 10.1098/rsfs.2023.0047

**Published:** 2023-12-15

**Authors:** Hussam Moammer, Jizhong Bai, Timothy L. M. Jones, Marie Ward, Caroyln Barrett, David J. Crossman

**Affiliations:** ^1^ Manaaki Manawa—The Centre for Heart Research, Department of Physiology, School of Medical and Health Sciences, Faculty of Medical and Health Sciences, Waipapa Taumata Rau / The University of Auckland, Park Road, Grafton, Auckland, New Zealand; ^2^ Department of Clinical Physiology, Faculty of Medicine, King Abdulaziz University, Jeddah, Saudi Arabia; ^3^ Division of Cardiology, Department of Medicine, University of Colorado Anschutz Medical Campus, Aurora, CO, USA

**Keywords:** transverse tubules, fibrosis, pirfenidone, heart failure, myocardial infarction

## Abstract

Transverse (t)-tubule remodelling is a prominent feature of heart failure with reduced ejection fraction (HFrEF). In our previous research, we identified an increased amount of collagen within the t-tubules of HFrEF patients, suggesting fibrosis could contribute to the remodelling of t-tubules. In this research, we tested this hypothesis in a rodent model of myocardial infarction induced heart failure that was treated with the anti-fibrotic pirfenidone. Confocal microscopy demonstrated loss of t-tubules within the border zone region of the infarct. This was documented as a reduction in t-tubule frequency, area, length, and transverse elements. Eight weeks of pirfenidone treatment was able to significantly increase the area and length of the t-tubules within the border zone. Echocardiography showed no improvement with pirfenidone treatment. Surprisingly, pirfenidone significantly increased the thickness of the t-tubules in the remote left ventricle of heart failure animals. Dilation of t-tubules is a common feature in heart failure suggesting this may negatively impact function but there was no functional loss associated with pirfenidone treatment. However, due to the relatively short duration of treatment compared to that used clinically, the impact of long-term treatment on t-tubule structure should be investigated in future studies.

## 1. Introduction

Transverse (t)-tubules are narrow invaginations of the sarcolemma that propagate the conduction of the action potential into the interior of the myocyte facilitating a coordinated intracellular Ca^2+^ release that triggers a forceful contraction through the process of excitation–contraction coupling [1,2]. Aberrant remodelling and loss of t-tubules is a prominent feature in both animal models of and human heart failure with reduced ejection fraction (HFrEF). Isolated cardiac myocytes from rodent [3,4], pig [5], and sheep [6] models of HFrEF have demonstrated prominent t-tubule remodelling, involving loss of the transverse elements, which is linked to discordant Ca^2+^ release. Moreover, imaging of living rat hearts and isolated muscle preparations (trabeculae) demonstrate that the observed remodelling reflects the structure of native tissue and is not merely an artefact of the isolation procedure [7,8]. Furthermore, experimental osmotic shock that leads to the disconnection of t-tubules from the cell surface in isolated myocytes and trabeculae results in impaired Ca^2+^ release and force production providing support for a causal link between t-tubule remodelling and loss of cardiac function [9,10]. Furthermore, the remodelling of t-tubules has been demonstrated in human HFrEF [11–13] and is correlated to contractile function [14,15].

The mechanisms of t-tubule remodelling in HFrEF is an area of active research with most studies focusing on the role of intracellular proteins involved in cardiac junction formation and stabilization [16–19]. We proposed an alternative hypothesis that fibrosis drives t-tubule remodelling based on our findings of increased collagen, particularly collagen VI within the lumen of the enlarged t-tubules of human HFrEF [20,21]. This hypothesis provides a mechanistic link between fibrosis [22] and aberrant Ca^2+^ handling [23] that are generally considered separate features in heart failure. Classically fibrosis is thought to impair cardiac relaxation a feature more strongly associated with heart failure with preserved ejection fraction (HFpEF) although a clinical study has identified a prognostic link to HFrEF and not HFpEF [24].

To test the hypothesis of fibrosis driving t-tubule remodelling we chose a rat myocardial infarction (MI) model of HFrEF that has previously been described to have both fibrosis and t-tubule remodelling [25,26]. This model was then treated with the anti-fibrotic pirfenidone, which is used clinically to treat idiopathic pulmonary fibrosis and has been shown to reduce fibrosis in humans with HFpEF [27]. Furthermore, pirfenidone has been shown experimentally to reduce fibrosis and improve cardiac function in a rat MI model of HFrEF [28,29]. By treating MI animals with pirfenidone, we hypothesized that we could reduce the fibrotic process within the failing heart and mitigate aberrant t-tubule remodelling.

## 2. Methods

All animal studies were conducted in male Wistar rats and were approved by and carried out following the guidelines of the Animal Ethics Committee of the University of Auckland (no. 002087). The rats aged 8–9 weeks were obtained from the Vernon Janson Unit, the University of Auckland, and housed in a room of constant temperature (22°C) with a 12 h light/dark cycle. They were given unrestricted access to standard chow and water. Regular monitoring of body weight, as well as food and water consumption, was performed. The following experiments were conducted in three groups: (1) sham, (2) MI and (3) MI treated with pirfenidone (MI + P) according to our previously published protocols as briefly described below. The MI + P group was administered pirfenidone in drinking water at 300 mg kg^−1^ day^−1^ [29] starting 7 days post MI.

### 2.1. Surgical procedures

Animals were anaesthetized with isoflurane anaesthesia (2% in oxygen) with continuous mechanical ventilation during surgery. A midline incision through the 4th intercostal space exposed and removed the pericardium, allowing direct access to the heart and left anterior descending coronary artery (LADC). MI was induced by tying off the left anterior descending coronary artery 2–3 mm from the origin using a 6–0 silk suture. In the sham groups, a suture was passed through the heart wall, but the LADC was not tied off. After the surgery, the lungs were reinflated, and the chest was sutured closed. For recovery after surgeries, animals were given prophylactic antibiotics (12.5 mg kg^−1^ enrofloxacin, Baytril; Bayer, Auckland, New Zealand) and analgesia (20 µg kg^−1^ buprenorphine Temgesic; Reckitt Benckiser, Auckland, New Zealand) and again 24 h later. After surgery, rats were returned to their home cages. A heating pad was placed under the cage for 24 h post-surgery. All rats were housed 2–4 per cage with water and food ad libitum in the dedicated animal room.

### 2.2. Echocardiography

To assess the cardiovascular function of the experimental rats, echocardiography was performed at 9 weeks post-MI surgery using a VEVO-Lazer-X high-resolution multimodal imaging system (FUJIFILM Visual Sonics Inc., Canada). In brief, the rat was anaesthetized (2–2.5% isoflurane in oxygen) until it could be handled easily and did not move during the imaging. The rat was then removed from the anaesthesia, and echocardiographic images were obtained to assess left ventricle (LV) function, including LV volume (parasternal long-axis view (PLAX)), LV systolic function (PLAX) and wall thickness of the LV chamber (short-axis view (SAX)). Analysis was performed by an observer blinded to the treatment groups.

### 2.3. Tissue collection and preparation

Subsequent to the final echocardiography, rats were anaesthetized, euthanized via decapitation, and hearts extracted. Following removal of fat, tissue and excess blood, hearts were weighed and perfused with a solution containing 20 mM butanedione monoxime to reduce energy expenditure [30] during removal of right ventricle (RV) trabeculae for force and Ca^2+^ measurement, described below.

Morphometric measurements were performed to record the wet weights of the body, heart, liver and lung, and the length of the tibia. Dry weights of the rat liver and lung were also obtained later. The heart was then transversely sectioned at the midventricular level. The top section apical to the ligation was fixed with 1% paraformaldehyde (PFA) in 0.1 M phosphate buffer (pH 7.4) for 1 h at 4°C, and then dehydrated using a series of graded alcohols before being snap frozen with OCT in 2-methylbutane using liquid nitrogen and stored in −80°C until processing for immunohistochemistry. The bottom section of the heart was then dissected into LV, septum and RV at smaller than 5 mm × 5 mm into cryo-tubes, snap-frozen in liquid nitrogen and stored in −80°C until processing for western blot analysis.

### 2.4. Force and intracellular Ca^2+^ measurements in trabeculae

Suitable trabeculae were removed from the RV and mounted in a temperature-controlled muscle chamber (Aurora Scientific, Canada), on the stage of an inverted microscope (Nikon Eclipse TE2000-U, Tokyo, Japan). Trabeculae were adjusted to optimal length and isometric force recorded (AE801, Kronex Technologies, USA) in response to electrical stimulation during superfusion with BDM-free Krebs–Henseleit solution containing 1 mM CaCl_2_ as previously described [31]. Trabeculae were then loaded with 10 µM fura-2 AM (Invitrogen, Thermo Fisher Scientific, USA) for ratiometric measurement of intracellular Ca^2+^ as previously described. Impaired calcium handling mechanisms in atrial trabeculae of diabetic patients [31]. The loading solution was replaced with Krebs–Henseleit buffer containing 1.5 mM CaCl_2_ and the muscle chamber temperature was raised to 37°C. Simultaneous measurements of isometric stress (force per trabeculae cross-sectional area) and intracellular Ca^2+^ (as fura-2 emitted fluorescence, at 340/380 excitation ratio) for experiments investigating a range of stimulation frequencies and extracellular Ca^2+^ concentrations. The force–frequency response at 0.2, 0.5, 1, 1.5, 2, 3, 5 and 7 Hz was obtained after steady-state was reached following each new frequency. Similarly, the response of trabeculae to extracellular Ca^2+^ concentration was obtained. Starting at 0.5 mM Ca^2+^, CaCl_2_ was added to the superfusate to raise the extracellular concentration in 0.5 mM increments up to an extracellular Ca^2+^ concentration of 3 mM. Steady-state measurements of stress and intracellular Ca^2+^ were then obtained. The stress and fluorescence data from 10 consecutive cycles were exported as text files to be averaged and analysed within a custom-written IDL program (Research Systems Inc., Boulder, CO, USA).

### 2.5. Western blot analysis

Western blot was performed as previously described [20]. Briefly, heart tissue was homogenized at 100 mg ml^−1^ in urea/thiourea extraction buffer (87% glycerol, 7 M urea, 2 M thiourea, 15 mM PBS at pH 8, 0.8% Triton X-100, 10 mM DTT, 5 mM EDTA and complete protease inhibitor (Roche). Homogenates were centrifuged (13 000*g*, 4°C, 10 min) and supernatants were retained for blotting. Samples were then mixed with loading buffer, incubated at 55°C for 15 min, and separated by SDS-PAGE (6 µl sample per lane, 4–15% Mini-PROTEAN TGX Stain-Free, Bio-Rad USA). Proteins were transferred onto PVDF membranes using the Trans-Blot Turbo Transfer System (Bio-Rad). Total protein concentration for blot normalization was determined using the Stain-Free method [32]. The gels were irradiated with UV and imaged before and post-transfer using the ChemiDoc MP System (Bio-Rad). After the transfer, the membranes were imaged, blocked in EveryBlot Blocking Buffer (no. 12010020, Bio-Rad) for 5 min with agitation, and incubated overnight at 4°C with a rabbit anti-collagen VI primary antibody (1 : 500, ab6588, Abcam) in the same blocking buffer. Following washes with Tris-buffered saline with 0.01% Tween 20 (TBST), membranes were incubated with an Alexa Fluor 647-conjugated goat anti-rabbit antibody (1 : 20 000, Thermo Fisher Scientific, USA) for 1 h at room temperature (RT). Blots were then imaged at optimum integration time using the above ChemiDoc MP System. For wheat germ agglutinin (WGA) staining, the blots were striped using a Restore Western Blot Stripping Buffer (no. 21059, Thermo Fisher Scientific) according to the manufacturer's instruction and re-probed overnight at 4°C with Alexa Fluor WGA 680 conjugate (1 : 200, Thermo Fisher Scientific). Protein band intensities were quantified using the Image Lab software (Bio-Rad) and normalized to total proteins of the same sample in the Stain-Free blots. Immunoblots were run separately for four repeats, and mean results were presented for analysis.

### 2.6. Immunohistochemistry and confocal microscopy

Immunohistochemistry was carried out on 10 µm thick tissue sections and mounted on poly-l-lysine coated no. 1.5 coverslips. Each heart yielded about five sections for subsequent immunohistochemistry analysis. The sections were incubated in PBS solution for 3 × 5 min, permeabilized with 1% Triton X-100 for 15 min, and blocked in Image-iT FX signal enhancer (Thermo Fisher Scientific) for 60 min all at RT. They were then incubated overnight at 4°C with a mouse monoclonal antibody to the ryanodine receptor (RyR; 1 : 100, cat. no. MA3-916, Thermo Fisher Scientific) for RyR labelling in a buffer solution containing 1% BSA/0.05% Triton X-100/0.05% NaN_3_/PBS. Following PBS washes, sections were exposed to secondary antibodies of goat anti-mouse Alexa Fluor 488 (1 : 200) and Alexa Fluor 594-conjugated WGA (1 : 200) for 2 h at room temperature. After secondary antibody treatment, sections were washed and mounted on slides using a 90% glycerol/PBS mounting solution. We used an LSM 800 Zeiss confocal microscope with a 63× 1.4 NA oil immersion objective for imaging. To satisfy Nyquist sampling requirement, 16-bit images were captured, at a pixel size of 85 nm. Cardiomyocyte images from the LV, RV and septum were obtained separately from each slide. Under 20× objective immunofluorescence microscopy, heart areas of interest (LV, RV, septum) were manually identified, and their coordinates were recorded using Zen Blue software (Zeiss). Subsequently, multiple high-resolution images were captured using the 63× objective, focusing on longitudinal cardiomyocyte sections to assess visible sarcomere periodicity.

### 2.7. Image analysis

ImageJ/FIJI was used to identify, rotate and crop individual cardiomyocytes from high-resolution confocal images. Cells were rotated (using bilinear interprolation) so the longitudinal centre of mass was vertical facilitating subsequent analysis of t-tubule angles, as described below. The heart regions sampled included the LV, septum, and RV. Within the MI hearts, the LV was further sub-sampled into the infarct border zone, and distal LV. Typically, 25 myocytes were analysed from each region in an individual heart. The selection of images from MI animals either untreated or treated with pirfenidone was unbiased as the observer remained blind to drug treatment, noting blinding to MI versus sham was not possible due to the presence of an infarct in the LV. Cells displaying curvature, inconsistent labelling, located at the image edge, or low contrast were excluded to minimize the influence of imaging artefacts.

**Figure 1.**
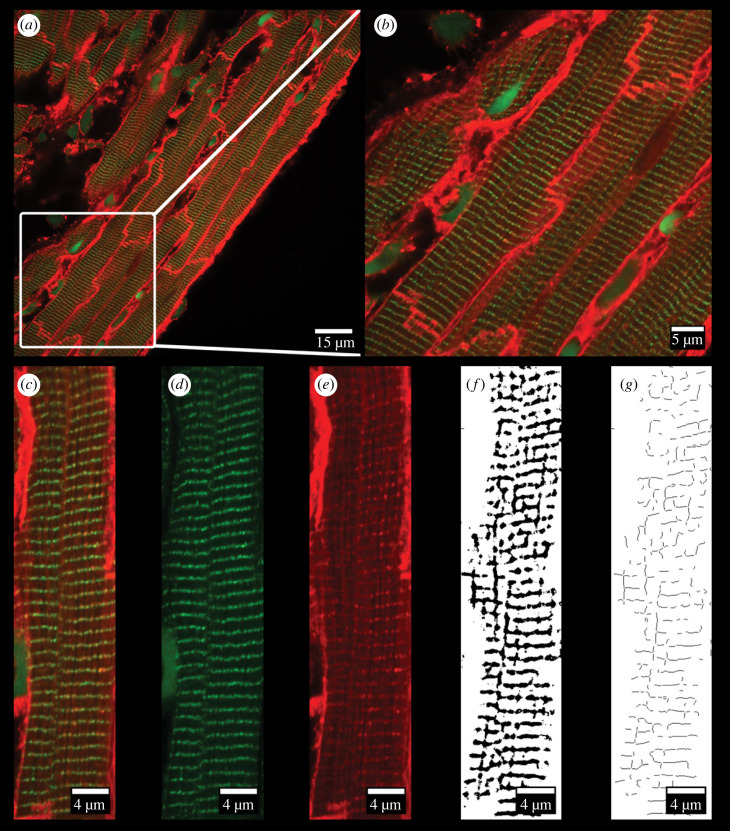
Segmentation of WGA and RyR labelled cardiac tissue. (*a*) Confocal image of cardiac tissue from the left ventricle labelled with WGA (red) and for RyR (green). (*b*) Enlargement of white box from (*a*). (*c*) Composite image of a selected single cardiomyocyte. (*d*) RyR labelling of the cardiomyocyte from (*c*). (*e*) WGA-594 labelling of the cardiomyocyte from (*c*). (*f*) Ilastik segmentation of t-tubules from WGA labelling. (*g*) Skeletonization of t-tubule mask.

The machine learning software, Ilastik was used to segment WGA labelling into both a cellular mask of the myocyte area and a mask of the t-tubules (figure 1). For segmentation, a training phase on a subset of cells was used to identify t-tubules by the use of a drawing tool to mark t-tubule structures and a further drawing tool to identify unwanted structures e.g. the extracellular matrix, cell wall, nucleus, and background labelling. After a satisfactory training phase, Ilastik was run on the entire dataset. The resulting binary images (figure 1*f*) were then exported from Ilastik for downstream analysis.

The sarcomeric regularity of both t-tubule and RyR labelling was assessed in the frequency domain, respectively referred to as T-power and R-power [33]. This analysis provides a measure of labelling regularity and was originally developed to asses t-tubule remodelling [3]. This analysis was carried out in ImageJ/FIJI and was automated by coding in the software's macro language circumventing the labour-intensive and error-prone manual analysis of thousands of images. This analysis involved converting the images of the t-tubules and RyR into the frequency domain using the fast Fourier transform (FFT) algorithm and measuring the height of the first harmonic peak by Gaussian curve fitting data from a 100-pixel line plot, similar to previous work [33]. Note, for RyR labelling the frequency analysis was carried out on the unprocessed cellular labelling. Due to low contrast of WGA t-tubule labelling the frequency analysis was carried out via the t-tubule mask.

The t-tubule binary was further analysed for t-tubule area, length, thickness and angle. T-tubule area was expressed as the per cent area of the t-tubule mask over the myocyte area. T-tubule length was calculated by first converting the t-tubule mask into a 1-pixel-wide skeleton using ImageJ/Fiji (figure 1*g*). T-tubule length was estimated for each cell by normalizing the length of the skeleton by cell area. The percentage of the t-tubule skeleton in transverse orientation was calculated using a custom algorithm written for the PYthon Microscopy Environment (PYME). The transverse elements were defined as tubules with an angle from 60−130° relative to the longitudinal mass of the cell.

### 2.8. Statistics

Our data analysis employed IBM SPSS Statistics 27 software. All data are expressed as mean ± SEM. Ejection fraction (EF), western blot and trabeculae data underwent GLM followed by Bonferroni *post hoc* analysis for group comparisons. A hierarchical mixed regression model was employed for the cellular measurements and data to account for the fact that cardiomyocytes from the same heart may be correlated. This approach allows for the consideration of within-subject correlations and improves the accuracy of the analysis. To further assess multiple comparisons in the *post hoc* analysis, the Dunn–Šidák correction (SIDAK) was applied.

## 3. Results

**Figure 2.**
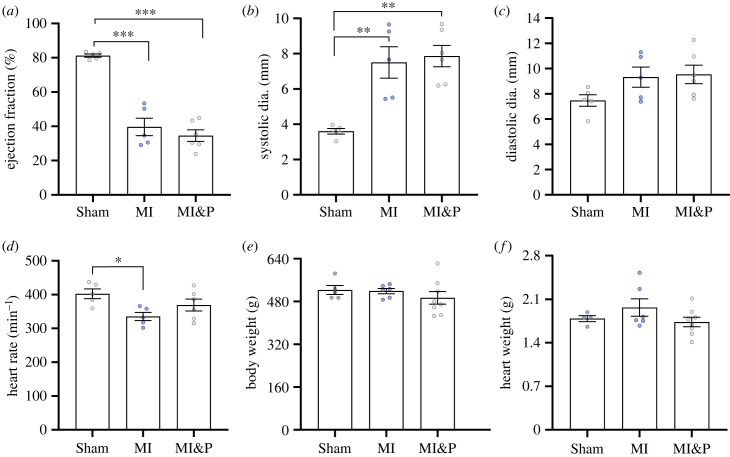
Echocardiography assessment of cardiac structure and function in sham-operated animals (sham) MI animals and MI animals treated with pirfenidone (MI&P). (*a*) EF. (*b*) Systolic diameter. (*c*) Diastolic diameter. (*d*) Heart rate. (*e*) Body weight. Statistical significance is indicated by * (*p* < 0.05) ** (*p* < 0.01) and *** (*p* < 0.001).

Echocardiography assessment at 9 weeks post-MI demonstrated a substantial and highly significant decrease in EF in the untreated MI group compared to sham-operated control animals (40 ± 5% versus 81 ± 1%, *p* < 0.001, figure 2*a*). The untreated MI animals showed a similar highly significant decline in fractional shortening (table 1). Moreover, the systolic diameter of the LV was substantially increased in the untreated MI group compared to sham animals (7.5 ± 0.8 mm versus 3.6 ± 0.2 mm, *p* < 0.01, figure 2*b*) but no change in diastolic diameter was observed. The MI group also showed a significant increase in systolic volume (table 1). Pirfenidone-treated MI animals showed no improvement in these parameters compared to MI animals but displayed a similar significant decrease in EF (35 ± 5% versus 81 ± 1%, *p* < 0.001, figure 2*a*) and fractional shortening (table 1). Similarly pirfenidone-treated MI animals also had a significant increase in systolic diameter (7.9 ± 1 mm versus 3.6 ± 0.2 mm, *p* < 0.01, figure 2*b*) and a significant increase in systolic volume (table 1) compared to sham animals. The MI group showed a modest but significant decrease in heart rate compared to sham animals (335 ± 11 versus 402 ± 11, *p* = 0.02, figure 2*d*). Echocardiography parameters of cardiac output, LV mass, diastolic volume and wall thickness showed no significant changes between the groups (table 1). No significant changes were found for the morphometrical measures that included body weight (figure 2*e*), heart weight (figure 2*f*), tibia length, liver weight, and lung weight (table 1).

**Table 1.** Summarized data of rat cardiac function and morphometrics for sham, myocardial infarction (MI) and myocardial infarction with pirfenidone (MI + P) treatment. LVAW-s, systolic left ventricular anterior wall thickness. LVAW-d, diastolic left ventricular anterior wall thickness. LVPW-s, systolic left ventricular posterior wall thickness. LVPW-d, diastolic left ventricular posterior wall thickness. Data are represented as mean ± s.e., *n* = 5–8 per group. Statistic differences relative to sham animals *p > 0.05, **p > 0.01, ***p > 0.001. No difference between MI and MI + P groups was found.

	sham	MI	MI + P
tibia length (mm)	51.2 ± 1.2	55.2 ± 0.5	56.6 ± 0.4
liver dry weight (g)	14.7 ± 0.6	16.1 ± 0.2	15.8 ± 0.3
lung dry weight (g)	1.65 ± 0.05	1.94 ± 0.04	1.71 ± 0.03
lung (wet/dry)	4.45 ± 0.05	4.01 ± 0.10	4.17 ± 0.04
systolic volume (µl)	55.5 ± 5.5	321 ± 79*	345 ± 57**
diastolic volume (µl)	302 ± 38	502 ± 92	530 ± 90
stroke volume (µl)	247 ± 33	181 ± 13	185 ± 43
fractional shortening (%)	51.5 ± 1.2	20.5 ± 2.9***	17.5 ± 1.9***
cardiac output (ml min^−1^)	100 ± 15	60.7 ± 5	63.2 ± 3.2
LV mass (mg)	1271 ± 201	1635 ± 314	1529 ± 134
LVAW-s (mm)	3.26 + 0.40	2.11 ± 0.69	1.63 ± 0.29
LVAW-d (mm)	2.16 ± 0.13	1.74 ± 0.42	1.41 ± 0.19
LVPW-s (mm)	3.19 ± 0.42	2.71 ± 0.42	2.76 ± 0.39
LVPW-d (mm)	2.02 ± 0.19	2.28 ± 0.47	2.34 ± 0.37

**Figure 3.**
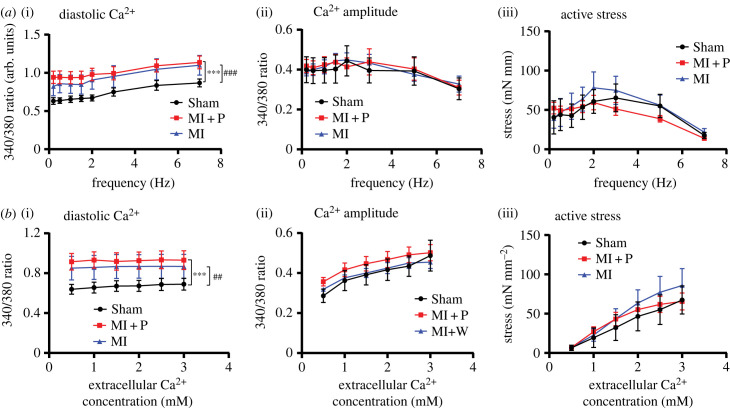
Trabeculae Ca^2+^ and stress dynamics in response to stimulation frequency (*a*) and extracellular Ca^2+^ (*b*). (i) Diastolic Ca^2+^, (ii) Ca^2+^ amplitude, (iii) diatostolic stress and (iv) active stress. Data represent mean ± s.e.m. for sham (*n* = 4) MI (*n* = 5) and MI + P (*n* = 6). Significance was determined by two-way ANOVA, with *p < 0.05, **p < 0.01 and ***p < 0.001 for group effect. * indicates a comparison between sham versus MI + P and # indicates a comparison between sham and MI.

To assess the effect of MI and pirfenidone treatment on the function of remote myocardium living RV trabeculae were isolated from the three groups for assessment of Ca^2+^ signalling and stress production. Two experimental protocols were used. The first assessed the stimulation-frequency response (figure 3*a*) and the second the extracellular-Ca^2+^ response (figure 3*b*). Trabeculae data analysis showed no difference between groups for Ca^2+^ transient amplitude, and active stress in response to both stimulation-frequency and extracellular-Ca^2+^ protocols. Diastolic Ca^2+^ (340/380 ratio, arbitrary units (a.u.)) was significantly elevated in RV trabeculae from both the MI group and the pirfenidone-treated MI group in comparison to sham animals for both the stimulation-frequency (0.92 ± 0.04, 1 ± 0.03, a.u., respectively versus 0.71 ± 21, a.u., *p* < 0.001) and extracellular-Ca^2+^ protocols (0.86 ± 0.04, 0.93 ± 0.03, a.u., respectively versus 0.67 ± 2, a.u., *p* < 0.01).

**Figure 4.**
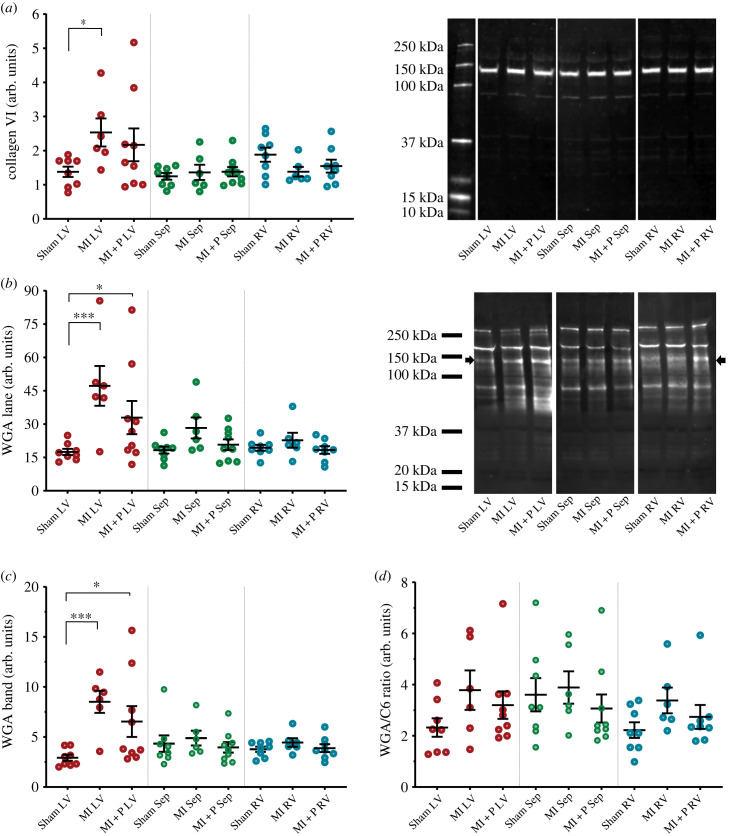
Western blot analysis of collagen VI and WGA positive proteins in sham-operated animals (sham) MI animals and MI animals treated with pirfenidone (MI + P). For each heart, three anatomical regions were assessed, left ventricle (LV) septum (Sep) and right ventricle (RV). (*a*) Collagen VI western data and exemplar blot with molecular weight ladder. (*b*) WGA total lane data and exemplar blot with indicative molecular weight ladder. The black arrow indicates the location of collagen VI. (*c*) WGA 140 kDa band data corresponding to collagen VI. (*d*) The ratio of the WGA band over collagen VI as an indicator of relative glycosylation. Statistical significance is indicated by * (*p* < 0.05) ** (*p* < 0.01) and *** (*p* < 0.001).

Western blot analysis demonstrated a significant increase in collagen VI expression in the MI LV compared to sham LV (2.5 ± 0.4 a.u. versus 1.4 ± 0.1 a.u., *p* = 0.01 figure 4*a*). In pirfenidone treated MI group the mean expression of collagen VI was lower but not significantly different from either the sham or MI animals. There was no change in collagen VI expression between the experimental groups in the septum or RV. Western blot analysis using WGA demonstrated a significant increase in total sialic acid containing proteins in the MI LV compared to sham LV (47 ± 9 a.u. versus 17 ± 1 a.u., *p* < 0.001, figure 4*b*). Although the pirfenidone treatment group had lower mean sialic acid levels, it was still significantly higher compared to sham animals (33 ± 11 a.u. versus 17 ± 1 a.u., *p* < 0.001, figure 4*b*). Analysis of the 140 kDa band corresponding to collagen VI (identified by overlaying the collagen VI and WGA blot images) also showed a significant increase in the MI LV compared to sham LV (8.5 ± 1.1 a.u. versus 2.9 ± 0.3 a.u., *p* < 0.001, figure 4*c*). Although the pirfenidone treatment had a lower mean level of the sialic acid band, this was also significantly higher compared to shams (6.5 ± 1.5 a.u. versus 2.9 ± 0.3 a.u., *p* = 0.02, figure 4*c*). Levels of total and 140 kDa band sialic acid levels were not significantly different among the groups in the septum or RV. Calculation of sialic acid to collagen VI ratio demonstrated no significant change in glycosylation between the groups in the LV, septum and RV (figure 4*d*).

**Figure 5.**
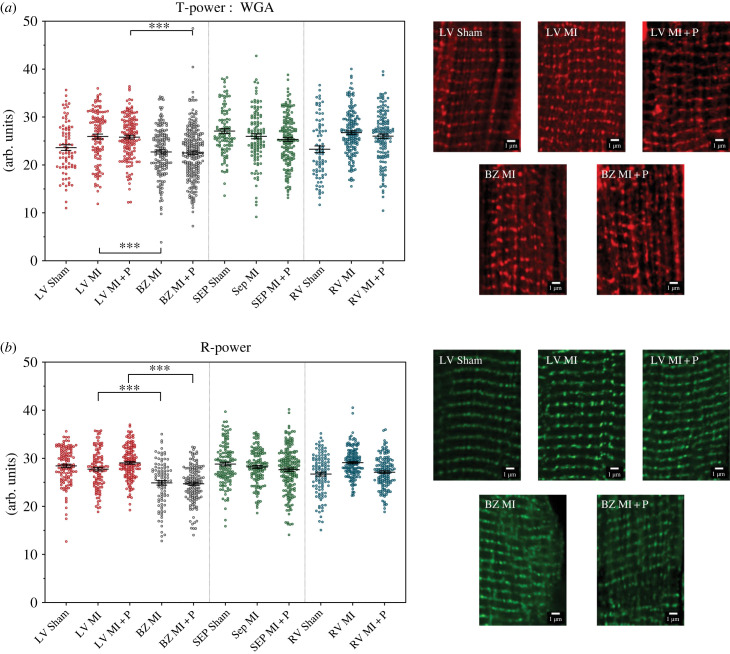
Frequency analysis of t-tubule (WGA) and RyR labelling in sham, MI, and MI + P groups. (*a*) Frequency analysis of t-tubule structure or T-power. For each heart, three anatomical regions were assessed, left ventricle (LV) septum (Sep) and right ventricle (RV). In the myocardial infarction groups, the LV was separated into two regions: remote LV (LV) and border zone LV (BZ). The data presented are mean and standard error of the mean. Each individual dot represents a single cardiomyocyte. There are 545 cells from five MI rats, 680 cells from five MI + P rats and 267 cells from five sham rats. Statistical significance is indicated by * (*p* < 0.05) ** (*p* < 0.01) and *** (*p* < 0.001). On the right side are exemplar confocal images of WGA labelling across the groups from the LV. (*b*) Frequency analysis of RyR labelling or R-power. Labels are the same as in (*a*). There are 497 cells from five MI rats, 514 cells from five MI + P rats and 359 cells from five sham rats. On the right are exemplar confocal images of RyR labelling across the groups from the LV.

Confocal microscopy imaging was used to assess the structure of cardiac myocytes in tissue sections labelled for t-tubules and RyR using WGA and antibodies, respectively. In sham animals, both t-tubules and RyR showed the expected striated sarcomeric labelling pattern. In the MI animals, there was disrupted t-tubular and RyR organization near the border zone compared to remote LV from the same animals (figure 5). A similar disruption was seen in the border zone of MI animals treated with pirfenidone. Within the remote LV, septum, and RV there were no discernible differences in either t-tubule or RyR labelling between the three animal groups. Images of individual cells were assessed in the frequency domain to assess the height of the frequency peak associated with the striated labelling pattern of the t-tubules (T-power) and the sarcoplasmic reticulum RyR (R-power). This analysis confirmed visual observation of the images and revealed a significant loss of sarcomere structure in the untreated MI border zone compared to the remote LV in both T-power (22.7 ± 0.4 a.u. versus 25.9 ± 0.5 a.u., *p* < 0.001, figure 5*a*) and R-power (24.9 ± 0.3 a.u. versus 27.4 ± 0.4 a.u., *p* < 0.001, figure 5*b*). A similar highly significant loss of sarcomere structure was observed in the pirfenidone-treated MI animals in the border zone compared to remote LV for both T-power (22.5 ± 0.4 a.u. versus 25.8 ± 0.4 a.u., *p* < 0.001, figure 5*a*) and R-power (24.7 ± 0.4 a.u. versus 29.0 ± 0.3 a.u., *p* < 0.001, figure 5*b*). There was no detectable change in sarcomeric frequency between MI and pirfenidone-treated MI animals in both T-power and R-power.

**Figure 6.**
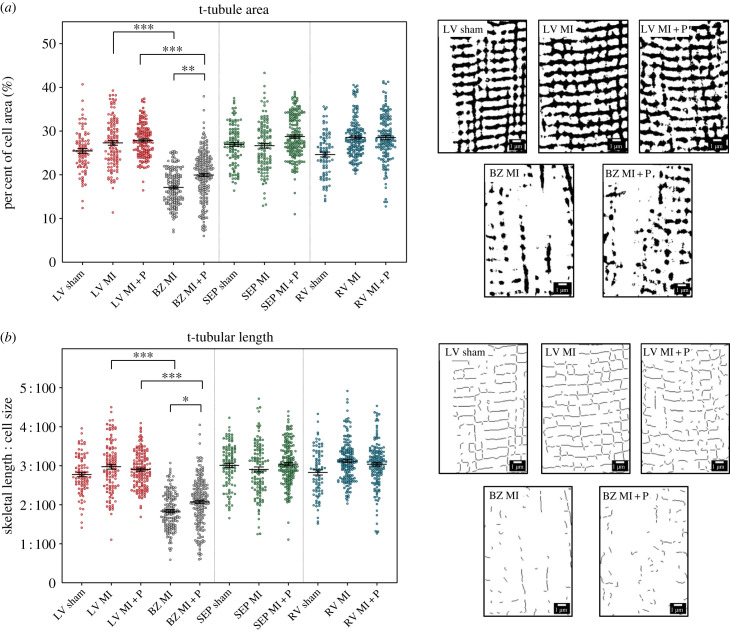
Analysis of t-tubule area and length in sham, MI, and MI + P groups. (*a*) Area of t-tubule mask. For each heart, three anatomical regions were assessed, left ventricle (LV) septum (Sep) and right ventricle (RV). In the MI groups, the LV was separated into a further two regions; remote LV (LV) and border zone LV (BZ). Data presented are mean and standard error of the mean. Each individual dot represents a single cardiomyocyte. There are 547 cells from five MI rats, 687 cells from five MI + P rats and 270 cells from five sham rats. Statistical significance is indicated by * (*p* < 0.05) ** (*p* < 0.01) and *** (*p* < 0.001). On the right side are exemplar images of the t-tubule area mask across the groups from the LV. (*b*) Length of the t-tubule skeleton. Labels and N are the same as in (*a*). On the right side are exemplar images of the t-tubule skeleton across the groups from the LV.

Analysis of the total t-tubule labelling area demonstrated a significant loss in the MI border zone compared to the remote LV in both untreated MI animals (26.5 ± 0.1% versus 15.9 ± 0.1% *p* < 0.001, figure 6*a*) and MI animals treated with pirfenidone (28.4 ± 0.1% versus 21 ± 0.1% *p* < 0.001, figure 6*a*). Notably there was a modest but significant increase in t-tubule area in the border zone in MI animals treated with pirfenidone compared to untreated MI group (21 ± 0.1% versus 15.9 ± 0.1%, *p* < 0.01, figure 6*a*). Analysis of total t-tubule length demonstrated a significant loss in the MI border zone compared to the remote LV in both untreated MI animals (2.9 ± 0.01% versus 1.7 ± 0.01%, *p* < 0.001, figure 6*b*) and MI animals treated with pirfenidone (2.9 ± 0.01% versus 2.1 ± 0.01%, *p* < 0.001, figure 6*b*). Moreover, there was a modest but significant increase in t-tubule length in the border zone in MI animals treated with pirfenidone compared to untreated MI group (2.1 ± 0.01% versus 1.7 ± 0.01%, *p* < 0.05, figure 6*b*).

**Figure 7.**
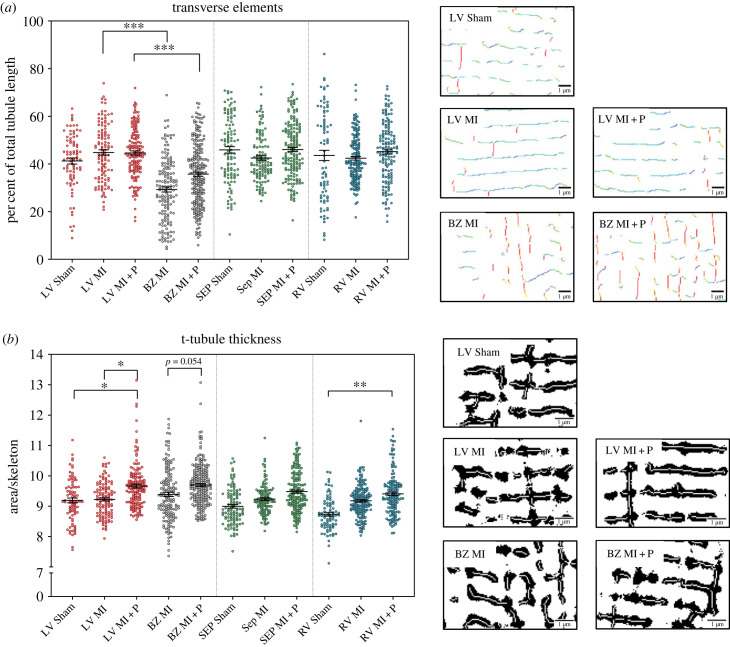
Analysis of t-tubule transverse angle and thickness ratio in sham, MI, and MI + P groups. (*a*) Transverse elements of the t-tubules. For each heart, three anatomical regions were assessed, left ventricle (LV) septum (Sep) and right ventricle (RV). In the myocardial infarction groups the LV was separated into a further two regions: remote LV (LV) and border zone LV (BZ). The data presented are mean and standard error of the mean. Each individual dot represents a single cardiomyocyte. There are 547 cells from five MI rats, 687 cells from five MI + P rats, and 270 cells from five sham rats. Statistical significance is indicated by * (*p* < 0.05) ** (*p* < 0.01) and *** (*p* < 0.001). On the right are t-tubule skeleton images, which are colour-coded according to tubular orientation, from blue representing transverse tubules at 0° through red representing axial tubules at 90°. On the right are images of segmented t-tubules (in black) overlayed by the t-tubule skeleton (in white). (*b*) t-Tubule thickness ratio (t-tubule mask/t-tubule length). Labels and N are the same as in (*a*). On the right side are exemplar images of the t-tubule area mask overlayed with the t-tubule skeleton across the groups from the LV.

An angle analysis of the t-tubule skeleton revealed a significant loss of the transverse elements of the t-system in the border zone relative to remote LV in both non-treated MI (29.3 ± 0.1 versus 44.8 ± 0.1 *p* < 0.001, figure 7*a*) and pirfenidone-treated MI animals (35.7 ± 0.09 versus 44.6 ± 0.09 *p* < 0.001, figure 7*a*) with no change between non-treated and pirfenidone treated MI. To further assess the structure of the t-tubules the binary masks were used to assess t-tubule thickness by dividing the t-tubule area mask by t-tubule length (figure 7*b*). This analysis showed no significant change in the t-tubule thickness ratio between the border zone and the remote LV in both treated and non-treated MI animals. However, the thickness ratio was significantly increased in the remote LV of the pirfenidone-treated MI animals compared to the remote LV of non-treated MI animals and sham LV (9.7 ± 0.04 versus 9.19 ± 0.08, and 9.38 ± 0.07 *p* < 0.05). Similarly, the thickness ratio increased, close to the significance cut-off, in the border zone LV of pirfenidone animals compared to the non-treated MI animals border zone (9.7 ± 0.04 versus 9.19 ± 0.08 *p* = 0.054). In both septum and RV, the mean t-tubules thickness increased in pirfenidone-treated animals but was only significant between the pirfenidone RV compared to sham RV (9.41 ± 0.06 versus 8.74 ± 0.06 *p* < 0.01).

## 4. Discussion

The results from this study demonstrate pirfenidone was able to partially reverse the loss of t-tubules that occurs within the border zone region of the infarct in MI heart failure. This was seen as an increase in total t-tubule area and an increase in t-tubule length. This supports the hypothesis that targeting fibrosis could provide a viable target to reverse t-tubule remodelling in heart failure [20]. However, pirfenidone was not able to mitigate cellular remodelling as reported by a loss of T-power, R-power, and transverse elements within the border zone. At the protein level, MI rats had significantly elevated levels of collagen VI within the infarcted LV compared to sham animals but these levels were not significantly decreased by pirfenidone treatment. However, collagen VI was not significantly greater in the LV of pirfenidone treated MI animals compared to the sham LV providing weak evidence of a possible reduction of collagen VI expression in the infarcted myocardium. These modest changes with pirfenidone treatment were not seen at the functional level. Echocardiography demonstrated a substantial change in cardiac structure and loss of cardiac function in the MI rats that included an enlarged systolic diameter, decreased EF and fractional shortening. However, pirfenidone treatment was not able to mitigate these changes suggesting modest if any benefit.

t-Tubule and RyR remodelling within the MI group was only found in the border zone adjacent to the infarct. Confocal microscopy of WGA labelled tissue sections revealed a significant loss of t-tubule regularity or T-power in the infracted border zone of MI hearts but not in the remote LV, septum and RV. Analysis of RyR labelling in the same sections showed a similar trend with a significant loss of R-power or regularity of RyR organization but only within the border zone. Analysis of t-tubule area, length, and transverse elements confirmed the FFT analysis showing a loss only within the infarct border zone. Loss of T-power, t-tubule area, t-tubule length and t-tubule transverse elements are all salient features of t-tubule remodelling found in both animal models and human HFrEF [4,7,11,15,20]. However, t-tubule remodelling was not a dominant feature in this rat model of MI heart failure as changes were limited to the infarcted border zone. This appears contrary to previous studies where notable remodelling of t-tubules have been documented on isolated myocytes from MI rats where presumably the majority of myocytes are remote to the border zone [15,34,35]. This discrepancy may relate to the myocyte isolation procedure inducing greater structure artefact in myocytes from fragile failing hearts. Alternatively, the disease process may be less severe in this work compared to previous studies.

The thickness of t-tubules was significantly increased in the remote LV of pirfenidone treated MI animals compared to remote LV in untreated MI animals and also the LV of sham operated animals. A similar increase in t-tubule thickness was seen in the border zone of pirfenidone treated animals compared to untreated animals that approached significance (*p* = 0.054). Within the RV, pirfenidone treated MI animals had a highly significant increase in t-tubule thickness compared to sham animals. A similar trend was found within the septum although this did not reach significance. These data indicate pirfenidone can elicit t-tubule dilation a feature that is characteristic of adverse t-tubule remodelling in both animal models and humans with heart failure [11,15,20,36]. However, in this study pirfenidone treatment resulted in no measurable loss in contractile performance in the LV as measured by echo or in RV trabeculae suggesting these changes are benign. This is further supported by a previous study showing improved EF in MI rats treated with pirfenidone [28]. However, both studies involve a short duration of pirfenidone treatment of 4–8 weeks. The implications of a longer-term pirfenidone treatment, as used clinically [37], are currently unclear and are of concern given this dilation that is reminiscent of t-tubule remodelling in heart failure. Pirfenidone has recently been shown to reduce extracellular matrix volume in the heart of patients with HFpEF [27]. It is highly germane then that the long-term effects of pirfenidone treatment on t-tubule structure be followed up in future studies.

Pirfenidone had no significant impact on diastolic Ca^2+^, Ca^2+^ amplitude, and active stress in RV trabeculae from MI animals. However MI-induced heart failure had no effect on rat RV trabeculae Ca^2+^ amplitude or active stress in both the stimulation frequency and extracellular-calcium protocols making it unlikely to detect an improvement in function. This is similar to a previous study of rat RV trabeculae approximately 17 weeks post MI that showed no change in force [38]. In comparison, another study showed a loss of force in rat RV trabeculae 24 weeks post MI [39] suggesting a comparatively mild heart failure phenotype in our study. Notably, we found diastolic Ca^2+^ was increased in RV trabeculae from treated, and untreated, MI animal groups for both the stimulation frequency and extracellular-Ca^2+^ protocols. A similar finding was observed in a previous study of isolated RV myocytes one week post coronary artery narrowing [40]. The increase in diastolic Ca^2+^ we observed is likely to be the result of leaky RyR as increased Ca^2+^ spark frequency has been reported in RV trabeculae from rat hearts approximately 17 weeks post MI [38]. In general, these studies indicate disturbance in diastolic Ca^2+^ is an early feature of cardiovascular disease after MI.

### 4.1. Limitations and future directions

The lack of a positive effect of pirfenidone on contractile function is in contradiction to a previous study on MI-induced heart failure [28] which found a modest improvement of ejection fraction in MI rats after only four weeks of pirfenidone. However, this study had a larger number of animals (*n* = 15 per group) than our study (5–6 per group) so likely had greater statistical power to detect a modest change in function.

T-tubule remodelling in this study was limited to only the border zone region adjacent to the infarct and although pirfenidone was able to reverse this remodelling the small size of the border zone may have reduced the ability to detect any associated functional changes. The small size of the border zone may also have affected the analysis of the western blot data where there was only weak evidence that pirfendone can reduce collagen VI expression in the infarcted LV. Note the LV samples in the westerns include remote LV, border zone, and infarct tissue.

Although there is some evidence collagen VI may contribute to t-tubule remodelling the role of other collagens cannot be excluded, particularly as collagen types I, III, and IV have also been found within the t-tubules [20]. Furthermore, WGA has been used to assess total fibrosis within the infarcted mouse heart indicating that other collagens were also present within the t-tubules in this study [41]. Moreover, in human HFrEF there is a substantial increase of sialic acid positive proteins, particularly associated with collagen VI [20]. This feature was also found within the infarcted LV in our study. However, unlike human heart failure, no increase in apparent glycosylation (WGA/collagen VI) was found in the MI rat heart. An increase in glycosylation ratio may be functionally important to the pathology of t-tubules [20] and may be a required feature in an animal model of t-tubule remodelling.

The increased thickness of the t-tubules after pirfenidone treatment documented in our study was likely underestimated. The average size of rat t-tubule diameter is 200 nm (*σ* ∼ 100 nm) based on super-resolution imaging with direct stochastic optical reconstruction microscopy (dSTORM) [42]. The resolution of confocal imaging used here is estimated at approximately 270 nm (based on fluorophore peak emission wavelength of 618 nm and NA of 1.4). This would result in substantial blurring of the t-tubules obscuring the actual difference in t-tubule thickness after pirfenidone treatment. Remodelling on the nanoscale could have important functional consequences. Fibrosis at the tissue scale is known to impact electrical conduction across the myocardium [43]. It is an intriguing proposition that at the nanoscale fibrosis could impair electrical conduction through the t-system.

## 5. Conclusion

Pirfenidone treatment was able to elicit a modest reversal of t-tubule remodelling within the border zone of the infarcted heart providing evidence for the hypothesis that fibrosis contributes to t-tubule remodelling and may provide a drug target for future heart failure therapies. There was weak evidence that pirfenidone reduced the elevated collagen VI levels in the infarcted LV, but the role of other collagens and sialic acid glycosylation in t-tubule remodelling cannot be excluded. The increased diameter of t-tubules after pirfenidone treatment is a concern given similarities to t-tubule dilation seen in heart failure. This finding should be followed up in future studies utilizing super-resolution microscopy to resolve changes in this cellular structure that is near to, or below, the resolution of the confocal microscope.

## Data Availability

We will provide data on request.
